# Preloaded Descemet membrane endothelial keratoplasty: meta-analysis of the eye bank prepared grafts transplanted in the last decade

**DOI:** 10.1038/s41433-026-04842-1

**Published:** 2026-09-08

**Authors:** Vito Romano, Maria Laura Passaro, Alessandro Ruzza, Stefano Ferrari, Diego Ponzin, John Lohmeier, Mohit Parekh

**Affiliations:** 1 Department of Medical and Surgical Specialties, Radiological Sciences, and Public Health, University of Brescia, Brescia, Italy; 2 Ophthalmic Unit, ASST Spedali Civili di Brescia, Piazzale Spedali Civili, Brescia, Italia; 3 Department of Eye and Vision Sciences, Institute of Life Course and Medical Sciences, University of Liverpool, Liverpool, UK; 4 International Center for Ocular Physiopathology, Fondazione Banca degli Occhi del Veneto Onlus, Venice, Italy; 5 Department of Research and Innovation, Rocky Mountain Lions Eye Bank, Aurora, CO USA

**Keywords:** Health services, Eye diseases

Preloaded Descemet membrane endothelial keratoplasty (pl-DMEK) involves ready-to-use eye-bank validated tissues. Clinical studies have demonstrated postoperative outcomes comparable to surgeon-prepared tissues, with endothelium-in (endo-in) and endothelium-out (endo-out) configurations [1]. pl-DMEK improves procedural standardization, surgical efficiency, and tissue utilization while lowering technical barriers to DMEK adoption [2, 3]. This meta-analysis consolidated clinical outcomes of pl-DMEK with endo-in and endo-out.

We followed PRISMA guidelines using PICOS framework. PubMed, Scopus, Web of Science, and Google Scholar were searched (June 2016–Aug 2025) using terms like ‘preloaded’, ‘eye-bank-prepared’, ‘ready-to-use’ and ‘DMEK’. Study design, sample, orientation, delivery device, media, and postoperative outcomes like endothelial cell loss (ECL), rebubbling rate, and visual acuity (ΔVA) at 3, 6, and 12 months were extracted. Statistical analyses were performed in R (v4.0.2). Rebubbling rates were pooled as proportions using a binomial generalized linear mixed model (GLMM) with random intercepts and Hartung–Knapp 95% confidence intervals (*p* < 0.05). Heterogeneity was assessed using Q, I^2^ and τ^2^ statistics, with maximum likelihood estimation of between-study variance. Subgroup differences were evaluated using χ², with leave-one-out approach and sensitivity analyses. Because variance data for ECL were inconsistently reported, postoperative ECL was summarized descriptively using weighted means. ΔVA was computed using random-effects model, stratified by orientation.

From 147 records, 19 studies (13 endo-in, 6 endo-out) comprising 1,420 eyes undergoing plDMEK met inclusion criteria. The average pooled rebubbling rate was 25.5% (95% CI, 19.7–32.3%). Rebubbling rates of 29.0% (95% CI, 22.4–36.6%) for endo-in and 19.9% (95% CI, 10.5–34.4%) for endo-out was recorded without significance (Fig. 1). Leave-one-out analyses demonstrated stable pooled estimates. For endo-in, heterogeneity remained substantial (I² 56–69%) with minimal influence from individual studies. For endo-out, exclusion of Rickmann et al. [4] reduced the pooled rebubbling to 14.9% (95% CI, 11.2–19.7%) and eliminated heterogeneity (I² = 0%). Sensitivity analysis excluding this study [4] showed a significant sub-group difference (*p* < 0.001), favoring endo-out (14.9%) over endo-in (29.0%). ECL at 3 months (weighted mean ECL) was 41.0% (228 eyes; endo-in) and 24.9% (358 eyes; endo-out). At 6 months, ECL was 36.9% (282 eyes) and 28.6% (558 eyes), respectively. At 12 months, ECL remained higher with endo-in (38.1%, 640 eyes) compared to endo-out (28.3%, 512 eyes) with no significant difference. Although trends suggest greater endothelial preservation with endo-out, inconsistent variance reporting and limited study numbers precluded formal meta-analysis. ΔVA improved substantially with pooled mean gains ranging from 0.4–0.5 logMAR to 0.53 logMAR (95% CI, 0.37–0.70) at 12 months, with no significant subgroup differences. High heterogeneity (I² 85%-93%) and small subgroup sizes limited definitive interpretation (Table 1).

**Fig. 1:**
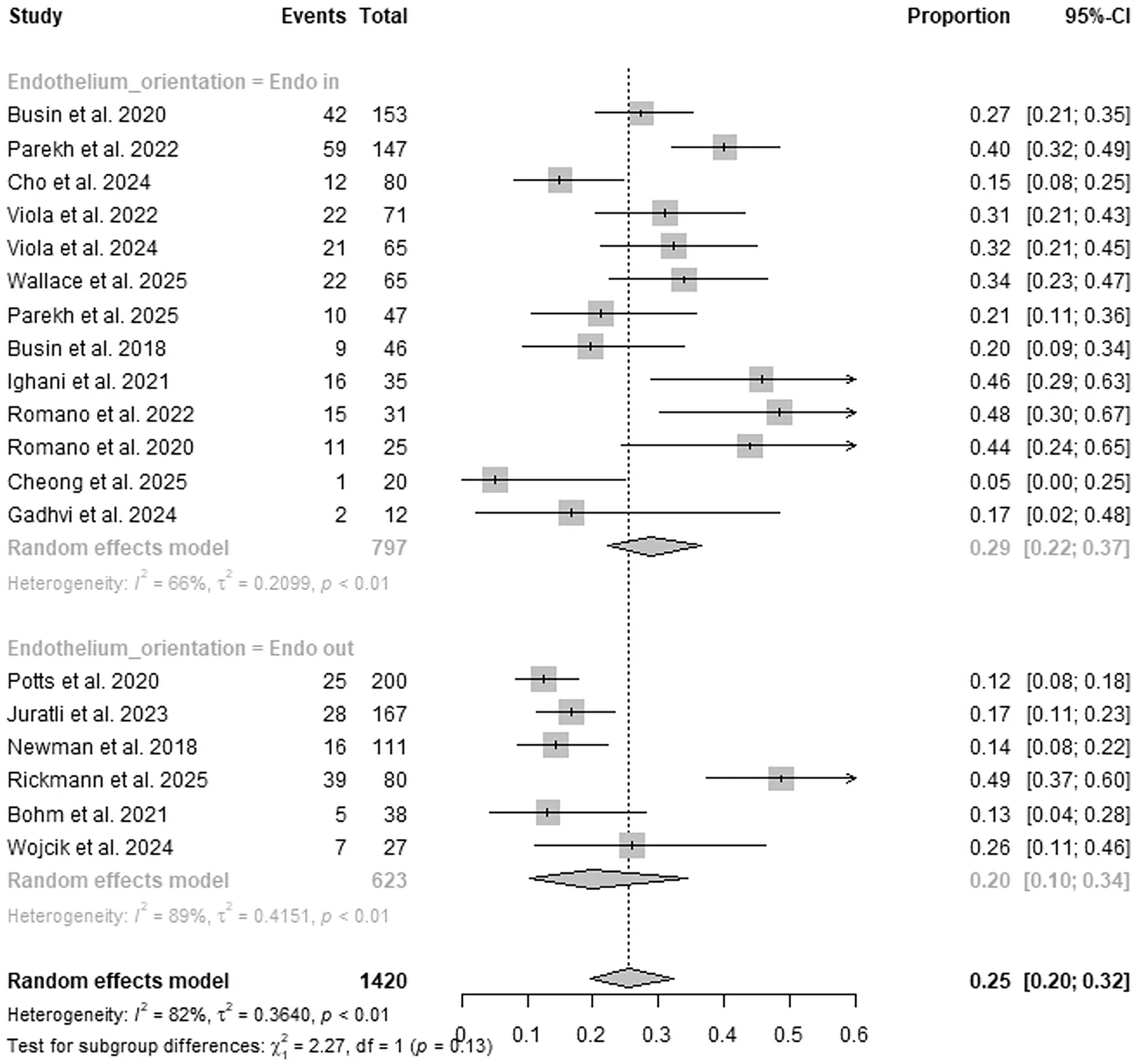
Forest plot of pooled rebubbling rates following preloaded DMEK with endo-in and endo-out techniques. Squares represent individual study estimates, with sizes proportional to study weight, and horizontal lines indicate 95% confidence intervals. The diamond indicates the pooled effect size for each subgroup and the overall estimate.

**Table 1:** Subgroup estimates were similar for endo-in and endo-out techniques, with no statistically significant difference.

Timepoint	Studies	Eyes (n)	Pooled ΔVA (95% CI)	I² (%)	Endo-in ΔVA (95% CI)	Endo-in I² (%)	Endo-out ΔVA (95% CI)	Endo-out I² (%)	*p*-value subgroup
3 months	4	285	0.42 (0.26–0.58)	84	0.47 (0.24–0.71)	0	0.37 (−0.64 to 1.39)	92	0.21
6 months	4	285	0.42 (0.28–0.56)	83	0.48 (0.27–0.68)	0	0.40 (−0.68 to 1.48)	93	0.35
12 months	7	504	0.53 (0.37–0.70)	94	0.61 (0.31–0.92)	95	0.42 (0.20–0.65)	85	0.08

Graft adhesion is multifactorial, influenced by storage duration, donor age, graft diameter, and combined procedures [5]. ΔVA improved substantially consistent with conventional DMEK results. ECL patterns align with prior reports consistent with our pooled estimates remaining comparable to surgeon-loaded DMEK at one year (~29–46%). Limitations include study heterogeneity, inconsistent reporting of clinical outcomes, and variability in donor and storage factors. The report highlights the clinical reliability of pl-DMEK; however, standardized protocols, long-term multicentre trials, and global collaboration remain essential priorities.

## Data Availability

The datasets generated during and/or analysed during the current study are available from the corresponding author on reasonable request.
